# Why be a part of the European Public Health Week? Marie Guichardon

**DOI:** 10.1093/eurpub/ckac129.286

**Published:** 2022-10-25

**Authors:** M Guichardon, A Schuffelen

**Affiliations:** EUPHA

## Abstract

What is the European Public Health Week? What's it's impact and why do you want to be part of it?

